# Dietary sodium intake in urban and rural Malawi, and directions for future interventions

**DOI:** 10.1093/ajcn/nqy125

**Published:** 2018-06-30

**Authors:** Josephine E Prynn, Louis Banda, Alemayehu Amberbir, Alison J Price, Ndoliwe Kayuni, Shabbar Jaffar, Amelia C Crampin, Liam Smeeth, Moffat Nyirenda

**Affiliations:** 1 Malawi Epidemiology and Intervention Research Unit, Lilongwe, Malawi; 2 Departments of Infectious Disease Epidemiology, London School of Hygiene and Tropical Medicine, London, United Kingdom; 3 Departments of Non-Communicable Disease Epidemiology, London School of Hygiene and Tropical Medicine, London, United Kingdom; 4 Department of International Public Health, Liverpool School of Tropical Medicine, Liverpool, United Kingdom; 5 Medical and Research Department, Dignitas International, Zomba, Malawi; 6 Department of Non-Communicable Disease, MRC/UVRI Uganda Research Unit, Entebbe, Uganda

**Keywords:** dietary sodium, hypertension, Malawi, Africa, cross-sectional, adult

## Abstract

**Background:**

High dietary sodium intake is a major risk factor for hypertension. Data on population sodium intake are scanty in sub-Saharan Africa, despite a high hypertension prevalence in most countries.

**Objective:**

We aimed to determine daily sodium intake in urban and rural communities in Malawi.

**Design:**

In an observational cross-sectional survey, data were collected on estimated household-level per capita sodium intake, based on how long participants reported that a defined quantity of plain salt lasts in a household. In a subset of 2078 participants, 24-h urinary sodium was estimated from a morning spot urine sample.

**Results:**

Of 29,074 participants, 52.8% of rural and 50.1% of urban individuals lived in households with an estimated per capita plain salt consumption >5 g/d. Of participants with urinary sodium data, 90.8% of rural and 95.9% of urban participants had estimated 24-h urinary sodium >2 g/d; there was no correlation between household per capita salt intake and estimated 24-h urinary sodium excretion. Younger adults were more likely to have high urinary sodium and to eat food prepared outside the home than were those over the age of 60 y. Households with a member with previously diagnosed hypertension had reduced odds (OR: 0.59; 95% CI: 0.51, 0.68) of per capita household plain salt intake >5 g/d, compared with those where hypertension was undiagnosed.

**Conclusions:**

Sodium consumption exceeds the recommended amounts for most of the population in rural and urban Malawi. Population-level interventions for sodium intake reduction with a wide focus are needed, targeting both sources outside the home as well as home cooking. This trial was registered at clinicaltrials.gov as NCT03422185.

## INTRODUCTION

High dietary sodium intake is a major risk factor for hypertension (1–4), which is itself the most important risk factor for death globally (5). Sodium consumption in most populations significantly exceeds the WHO’s recommended amounts of <2 g/d (equivalent to 5 g salt) in adults (2, 6). Several countries in sub-Saharan Africa (SSA), including Malawi, report a high prevalence of hypertension; however, data on sodium consumption from the region are scarce (6, 7). Understanding sodium consumption in Africa may be of particular importance because it is suggested that its populations have an increased sensitivity to the hypertensive effects of sodium (8).

Quantifying dietary sodium intake is challenging. Analysis of 24-h urinary sodium is the gold-standard measurement method, as the large majority of dietary sodium is renally excreted (9, 10). However, it is resource-intensive and places considerable burden on research participants (11), with potential negative impacts on response rates and sample quality (12). Spot urine sampling is a less burdensome tool for assessing population-level sodium intake but individual variation in urinary sodium concentration is too high for this measure to be predictive of individual-level sodium intake (12, 13). Self-reported salt consumption is an alternative method, although also prone to measurement error owing to the challenge of estimating the amount of salt used in food prepared away from the home or in processed foods, the amount of salt used in cooking, and variation in how much salt is retained in food after cooking (10, 14).

The majority of sodium intake in many high-income settings comes from processed foods, which historically have not been a substantial part of the diet in SSA (10, 15, 16). However, intake of processed foods is increasing rapidly in SSA, particularly in urban areas (16, 17), and this may be affecting the sources of dietary sodium in African populations. Evidence suggests higher dietary sodium intake in urban compared with rural populations in SSA (7) and higher hypertension prevalence in some urban populations compared with rural ones (18–22), although this difference has not been observed consistently (23).

Our study explored the extent of daily dietary sodium consumption and its variation between urban and rural populations within Malawi, which is a predominantly rural SSA country experiencing rapid urbanization (24).

## METHODS

### Study design and population

The study was a large cross-sectional population-based survey in 2 areas of Malawi: a rural area within Karonga District that is a well-established Health and Demographic Surveillance Site (total population of 40,000 individuals, including 15,806 adults aged ≥18 y); and Area 25 of Lilongwe, the capital city (total population of 66,000 individuals, including 24,367 adults aged ≥18 y). The rural area is a mainly subsistence economy, whereas the urban area has a mix of private- and public-sector employment. Data were collected from May 2013 to February 2015.

The survey assessed the population burden of hypertension, diabetes, lipid disorders, and their risk factors, and the primary outcomes have previously been published (22). The design and the standardized protocols and equipment used are also described in detail elsewhere (25). In brief, every household in both areas was visited by a field team, and every adult aged ≥18 y was eligible. Interview data including medical history were collected through the use of structured questionnaires, and anthropometric measurements, blood pressure, and blood samples were taken. On a subsample of participants, we collected spot morning urine samples: specifically, from all participants recruited consecutively on a Monday or a Wednesday until the predecided subsample size was reached. Individuals who were absent at the initial visit were revisited twice more before being categorized as missed.

### Measurements and variables

Daily sodium intake was calculated in 2 ways: first, by estimating a minimum salt intake measured as the per capita consumption of household plain salt used, and then estimating a total sodium intake from an estimated 24-h urinary sodium excretion based on analysis of spot urine samples in a subset of participants. A conversion factor of 2.5 for sodium to NaCl salt was utilized, as per WHO guidelines (2), so that 1 g Na is equivalent to 2.5 g salt.

Per capita consumption of plain salt used in the household was estimated by a novel approach, which was chosen given the known limitations of the standard methods of dietary sodium measurement. This population is known to consume predominantly nonprocessed foods, mainly prepared within the home, and therefore household plain salt may be the sodium source most amenable to change. Participants were asked for how many days a standardized 50-g bag of salt (the quantity typically purchased; an example of this was shown as a visual cue) would last in their household. We calculated grams of salt consumed per day per adult based on responses to this, and on the number of adults and children living in each household. The calculation assumed that adults would eat 4 times the volume of food prepared with the salt that younger children would (aged 0–5 y), and twice as much as older children (aged 5–17 y). Participants were also asked how frequently they ate food prepared away from home to assess whether the salt consumed in the household represented the bulk of their salt consumption.

Information on sociodemographic, health, and lifestyle factors was also collected via survey and is described in detail elsewhere (22, 25). In brief, data on an individual's educational attainment were categorized as illiterate/no formal education, attended Standard 1–5, attended Standard 6–8, or secondary/tertiary, with information on parental education grouped into the same categories, and defined as the highest educational level attained by either parent. Occupation data were collected as precoded categories: government employee, nongovernment employee, subsistence farmer/fisherman, self-employed, student, at home doing housework, unemployed (but able), unemployed (unable), and retired. In this analysis, government and nongovernment employee categories were combined, and unemployed (but able), unemployed (unable), and retired were combined, due to data sparsity in these groups. Household wealth was measured with a household possession score, which awarded a score of 1–10 based on the value of possessions that occupants owned, which was divided into fifths for the analysis.

Weight and height were measured twice on each participant, following removal of shoes and outer clothes, through the use of calibrated Seca scales, stadiometers, and flexible tape-measures. The mean of the 2 measurements was used in analyses. Based on the height and weight measures, BMI was calculated in kg/m^2^ and grouped according to standard cutoffs: <18.5, 18.5–24.9, 25–29.9, and ≥30.

Blood pressure was measured on the right upper arm, 3 times in a seated position after 30 min of inactivity and with 5 min of rest in between measurements, with the use of portable electronic devices (OMRON Healthcare Co., Ltd. HEM-7211-E, Model M6). The blood pressure used in the analysis was an average of the second and third readings. Hypertension was defined as systolic blood pressure ≥140 mm Hg and/or diastolic blood pressure ≥90 mm Hg, or self-report of current antihypertensive medication. Anybody who reported a previous diagnosis of hypertension but had a normal blood pressure on measurement and was not on any medications was not categorized as having hypertension.

Urine was tested for sodium, potassium, and creatinine. Urine sodium and potassium were assayed via the crown ether membrane electrode method, and urine creatinine was tested via the kinetic Jaffé method. A Beckman Coulter AU480 analyzer was used for the urinary analysis. Urine electrolyte data were used to estimate the 24-h urinary sodium output with the INTERSALT equation. This is {23 × [25.46 + (0.46 × spot Na) – (2.75 × spot Cr) – (0.13 × spot K) + (4.10 × BMI) + (0.26 × age in y)]} for men, and {23 × [5.07 + (0.34 × spot Na) – (2.16 × spot Cr) – (0.09 × spot K) + (2.39 × BMI) + (2.35 × age) – (0.03 × age^2^)]} for women, where sodium (Na), creatinine (Cr), and potassium (K) are in mmol/L (26).

### Ethical approval and funding

Ethical approval was granted by the Malawi National Health Sciences Research Committee protocol number #1072, and the London School of Hygiene and Tropical Medicine Ethics Committee protocol number #6303.

### Statistical analysis

We investigated the distribution of salt intake by sociodemographic and health-related behavioral risk factors stratified by location (urban/rural) and sex: proportions are expressed with 95% CIs. Pearson's correlation tests were used to assess correlation between reported per capita daily plain salt consumption and estimated daily salt intake based on spot urinary electrolyte analysis. The effect of a prior diagnosis of hypertension on sodium intake at individual and household levels was estimated via logistic regression analysis, with adjustment for potential confounders. In this analysis, age groups were combined into groups of <40, 40–60, and >60 y, owing to data sparsity at lower ages in this subgroup. Stata version 14.0 (StataCorp, College Station, TX) was used. Biologically impossible estimated total daily sodium intake for 1 (0.001%) participant was excluded from these analyses.

## RESULTS

Of 30,365 individuals included in the survey, 29,074 (96%) had household-level salt consumption calculated: 13,612 from the rural site and 15,462 from the urban site. The missing salt data were in most cases due to missing data on number of household members, precluding the calculation of per capita household salt intake. BMI data were missing in 3.7% of individuals, mainly as it was not calculated for pregnant women. No other variable was missing >1% of data. Urinary electrolytes were measured in 1051 rural participants and 1027 urban participants.

**FIGURE 1:**
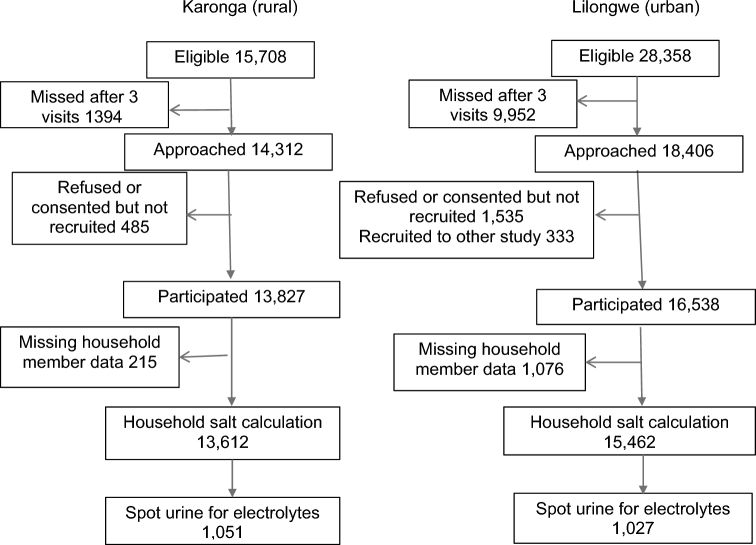
Flow chart of participant recruitment.

In the rural area, 8.9% of residents were missed during the data collection, compared to 35.1% in the urban area, and in both sites men were more likely to be missed than women. A flow chart of participant recruitment is shown in **Figure 1**.

### Participant characteristics

The baseline characteristics of participants are summarized in **Table 1**. Urban participants were significantly more likely than rural participants to be aged <30 y (*P* < 0.001), to have completed secondary education (*P* < 0.001), to live in households with a wealth score >3 (*P* < 0.001), and to have a BMI of >25 (*P* < 0.001) when controlled for sex. Most participants were in the healthy BMI range (18.5–24.9), although BMI varied by sex and study site, with only 51.5% of urban women within the healthy range. In almost all households, in both rural and urban areas, most of the food consumed was prepared within the home; however, individuals who were younger, male, and urban residents were more likely to regularly eat food prepared away from the home: 19.3% (95% CI: 18.3%, 20.3%) of urban men reported eating food prepared outside of the home sometimes or often, compared with 11.0% (95% CI: 10.5%, 11.7%) of urban women, 6.7% (95% CI: 6.1%, 7.5%) of rural men, and 5.7% (95% CI: 5.2%, 6.3%) of rural women.

**TABLE 1:** Baseline characteristics of the study population by study site^1^

	Rural		Urban	
	Women	Men	Women	Men
	*n* (%)	*n* (%)	*n* (%)	*n* (%)
Age, ^2^ y				
<30	2888 (36.7)	2186 (38.1)	5142 (50.9)	2689 (50.3)
30–39	1949 (24.7)	1393 (24.3)	2860 (28.3)	1321 (24.7)
40–49	1221 (15.5)	892 (15.6)	1068 (10.6)	646 (12.1)
50–59	832 (10.6)	568 (9.9)	572 (5.7)	342 (6.4)
≥60	987 (12.5)	696 (12.1)	456 (4.5)	350 (6.5)
Education level				
Illiterate/literate but no formal education	1233 (15.7)	358 (6.2)	935 (9.3)	153 (2.9)
Standard 1–5	611 (7.8)	369 (6.4)	526 (5.2)	178 (3.3)
Standard 6–8	4014 (51.0)	2377 (41.5)	2351 (23.3)	779 (14.6)
Secondary or tertiary	2019 (25.6)	2631 (45.9)	6299 (62.3)	4241 (79.3)
Occupation				
Employed	189 (2.4)	506 (8.8)	1550 (15.3)	2007 (37.5)
Subsistence farmer/fisherman	5206 (66.1)	3319 (57.9)	46 (0.5)	30 (0.6)
Self-employed	1019 (12.9)	836 (14.6)	1812 (17.9)	1084 (20.3)
Student	331 (4.2)	672 (11.7)	1219 (12.1)	1164 (21.8)
At home doing housework	750 (9.5)	68 (1.2)	4426 (43.8)	339 (6.3)
Unemployed/retired/other	382 (4.9)	334 (5.8)	1058 (10.5)	727 (13.6)
Household wealth score				
1 (poorest)	2309 (29.3)	1510 (26.3)	1271 (12.6)	544 (10.2)
2	1958 (24.9)	1490 (26.0)	1100 (10.9)	581 (10.9)
3	1710 (21.7)	1284 (22.4)	2009 (19.9)	979 (18.3)
4	1379 (17.5)	1050 (18.3)	2070 (20.5)	1103 (20.6)
5 (wealthiest)	521 (6.6)	401 (7.0)	3661 (36.2)	2144 (40.1)
BMI,^3^ kg/m^2^				
<18.5	523 (7.1)	559 (9.8)	386 (4.0)	376 (7.0)
18.5–24.9	4836 (65.7)	4649 (81.3)	4940 (51.6)	3982 (74.4)
25–29.9	1436 (19.5)	454 (7.9)	2523 (26.3)	787 (14.7)
≥30	565 (7.7)	58 (1.0)	1729 (18.1)	205 (3.8)

^1^Excluding those with missing household per capita salt intake data.

^2^16 missing values (13 urban women, 3 urban men).

^3^1066 missing values (517 rural women, 15 rural men, 533 urban women, 1 urban man).

### Estimated sodium consumption

**Table 2** shows the proportion of participants identified as likely to be consuming more than the recommended 5 g salt/d (or 2 g Na) as determined by the 2 methods used in this study, by sex and by urban or rural location. Examining reported household salt usage, 52.8% (95% CI: 52.0%, 53.7%) of rural and 50.1% (95% CI: 49.3%, 50.9%) of urban participants were residing in households with per capita plain salt intake of >5 g/d, equivalent to 2 g Na. In contrast, using spot urinary sample analysis to estimate total daily sodium consumption, 90.8% (95% CI: 88.9%, 92.4%) of rural and 95.9% (95% CI: 94.1%, 97.0%) of urban participants had an estimated salt intake of >5 g/d (or 2 g Na), which can be seen in **Table 3**. Among rural participants, 3.0% (95% CI: 2.2%, 4.3%) had an estimated total sodium consumption of >10 g salt (or 4 g Na)/d, compared with 4.5% (95% CI: 3.4%, 5.9%) of urban participants (data not shown in tables). Pearson's correlation coefficient between reported per capita purchased daily plain salt consumption and estimated daily salt intake based on spot sample urinary electrolyte analysis was –0.03 for rural participants and +0.03 for urban participants.

**TABLE 2:** Percentage of the population living in households with per capita plain salt intake of >5 g/d, and of individuals with estimated total salt intake >5 g/d based on urinary analysis

	Rural				Urban			
	Individuals living in households with per capita plain salt intake >5 g/d		Individuals with estimated total salt intake >5 g/d^1^		Individuals living in households with per capita plain salt intake >5 g/d		Individuals with estimated total salt intake >5 g/d^1^	
	Women	Men	Women	Men	Women	Men	Women	Men
*n* % (95% CI)	7877	5735	579	472	10,111	5351	710	316
	53.6 (52.5, 54.7)	51.8 (50.5, 53.1)	89.5 (86.9, 91.7)	92.4 (89.6, 94.5)	50.9 (49.9, 51.8)	48.8 (47.4, 50.1)	95.4 (93.5, 96.7)	97.2 (94.6, 98.5)

^1^INTERSALT equations used to estimate 24-h urinary sodium excretion (milligrams per day).

**TABLE 3:** Proportion of participants with ≥2 g Na (or 5 g salt) intake/d and the mean sodium intake per day in the rural and urban study sites, estimated from urinary electrolyte analysis by age and sex^1^

	Rural			Urban		
	Women	Men	Total	Women	Men	Total
	% (95% CI)	% (95% CI)	% (95% CI)	% (95% CI)	% (95% CI)	% (95% CI)
*n*	579	472	1054	710	316	1026
Age, y						
<30	92.3 (87.6, 95.3)	89.4 (83.2, 93.5)	91.1 (87.6, 93.7)	94.8 (91.7, 96.8)	98.1 (94.2, 99.4)	95.9 (93.7, 97.4)
30–39	91.6 (86.0, 95.0)	89.2 (82.5, 93.5)	90.5 (86.4, 93.4)	97.3 (94.0, 98.8)	98.3 (89.1, 99.8)	97.5 (94.8, 98.8)
40–49	96.7 (90.2, 98.9)	92.2 (83.7, 96.5)	94.6 (90.0, 97.2)	98.9 (92.8, 99.9)	95.5 (83.6, 98.9)	97.8 (93.4, 99.3)
50–59	92.3 (83.9, 96.5)	100 (*n* = 62)	95.7 (90.8, 98.1)	93.6 (84.0, 97.6)	97.0 (81.4, 99.6)	94.7 (88.0, 97.8)
≥60	61.3 (48.7, 72.5)	98.4 (89.4, 99.8)	79.8 (71.9, 86.0)	78.6 (59.8, 90.0)	91.7 (72.1, 97.9)	84.6 (72.1, 92.1)
Overall	89.5 (86.7, 91.7)	92.4 (89.6, 94.5)	90.8 (88.9, 92.4)	95.4 (93.5, 96.7)	97.2 (94.6, 98.5)	95.9 (94.1, 97.0)

^1^INTERSALT equations used to estimate 24-h urinary sodium excretion (milligrams per day).

The proportion with total daily sodium intake of >2 g (equivalent to 5 g salt) based on urinary electrolyte analysis is shown in Table 3, by age and sex. Sodium intake was found to be slightly higher in younger adults, men, and those living in urban areas. Mean sodium intake was 2.7 g in the rural area (equivalent to 6.8 g salt) and 2.9 g in the urban area (equivalent to 7.1 g salt).

### Effect of a prior diagnosis of hypertension on sodium intake

Households with members known to have hypertension prior to the survey had lower odds of per capita daily household plain salt intake >5 g (equivalent to 2 g Na) than those whose household members had undiagnosed hypertension prior to the survey, with ORs of 0.59 (95% CI: 0.51, 0.68) in rural and 0.90 (95% CI: 0.80, 1.02) in urban areas, controlling for age, sex, household wealth score, education, occupation, and a known parental history of hypertension. The same pattern of lower odds of salt intake >5 g existed when using urinary sodium excretion as a proxy for total sodium intake, with ORs of 0.56 (95% CI: 0.21, 1.51) in the rural population and 0.20 (95% CI: 0.04, 0.95) in the urban population, controlling for the same factors. Similarly, individuals with known hypertension appeared to have lower odds of salt intake >5 g/d (equivalent to 2 g Na) than those with unknown hypertension: 0.23 (95% CI: 0.04, 1.17) and 0.38 (95% CI: 0.08, 1.88) in the rural and urban areas, respectively, controlling for the same factors, although the CIs cross the null value. This is summarized in **Table 4**.

**TABLE 4:** Effect of a previously known diagnosis of hypertension on salt consumption, compared with unknown hypertension

	Proportion of participants with salt intake >5 g/d		Unadjusted		Adjusted^1^	
	Undiagnosed hypertension	Diagnosed hypertension	OR (95% CI)	*P* value	OR (95% CI)	*P* value
Proportion of participants with per capita household plain salt intake >5 g/d, and comparison of participants from households with ≥1 member with known hypertension to those with members with undiagnosed hypertension^2^						
Rural	40.8% (917/2245)	27.3% (374/1370)	0.54 (0.47, 0.63)	<0.001	0.59 (0.51, 0.68)	<0.001
Urban	43.4% (924/2130)	38.4% (1006/2622)	0.81 (0.72, 0.91)	<0.001	0.9 (0.80, 1.02)	0.1
Proportion of participants with estimated total salt intake >5 g/d^3^ and comparison of participants from households with ≥1 member with known hypertension to those with members with undiagnosed hypertension^2^						
Rural	94.1% (143/152)	84.0% (89/106)	0.33 (0.14, 0.77)	0.01	0.56 (0.21, 1.51)	0.25
Urban	98.2% (108/110)	92.1% (197/214)	0.21 (0.05, 0.95)	0.04	0.20 (0.04, 0.95)	0.04
Proportion of participants with estimated total salt intake >5 g/d,^3^ and comparison of participants with previously diagnosed hypertension to those with undiagnosed hypertension						
Rural	96.1% (74/77)	79.7% (55/69)	0.16 (0.04, 0.59)	0.01	0.23 (0.04, 1.17)	0.08
Urban	95.2% (60/63)	88.4% (99/112)	0.34 (0.09, 1.25)	0.11	0.38 (0.08, 1.88)	0.24

^1^Adjusted for age (<40 y, 40–60 y, >60 y), sex (male/female), household wealth score (measured as a score based on number of specific items owned by occupants, and categorized into fifths), education (no formal education, attended classes Standard 1–5, attended classes Standard 6–8, secondary/tertiary), occupation (employed, subsistence farmer/fisherman, self-employed, student, at home doing housework, unemployed/retired/other), and parental education level (defined as the highest level of education attained by either parent, with the same categories as participant education level).

^2^Excluding participants from households with nobody classified as hypertensive at the time of the survey.

^3^Estimated total salt intake calculated with the INTERSALT equations to estimate 24-h urinary sodium excretion (mg/d).

## DISCUSSION

In this large population-based study we found sodium intake was substantively higher than the recommended WHO limit of 2 g/d for the majority of adults in both rural and urban populations. Working-age, urban, and male participants were more likely to regularly eat food outside of the home, and to have an estimated total sodium intake of >2 g/d.

Reported household per capita plain salt intake alone exceeded the recommended daily limit of 5 g (or 2 g Na) for ∼50% of individuals in both the rural and urban study sites, and estimated total sodium intake was above the recommended limit for >90% of individuals based on urinary electrolyte analysis. Although sodium consumption is high in both urban and rural areas, the urinary analysis suggests that urban residents were slightly more likely to consume >2 g Na (5 g salt)/d. Further qualitative research in the same populations also found that urban residents commonly use high-sodium food additives in home cooking in preference to plain salt (H Namadingo, Malawi Epidemiology and Intervention Research Unit, Lilongwe, Malawi, personal communication, 2017) and, as expected, have a much higher consumption of processed foods (S Parkinson, London School of Hygiene and Tropical Medicine, London, United Kingdomm, personal communication, 2017).

Data were not available on sodium content in common ingredients such as flavor enhancers or stock cubes, in purchased foods (including bread), or in food prepared away from the home. Hence, the household-level consumption captured by the questionnaire provides only a minimum estimate of sodium intake, whereas the urinary sodium analysis represents all consumed sodium. The lack of correlation between reported home salt usage and urinary sodium excretion highlights the challenges in consistently capturing sodium intake across populations with varying eating patterns. Even in a population where most food is prepared at home, household per capita plain salt intake alone does not give a full picture of total sodium intake.

The mean sodium intakes estimated from the urinary analysis of 2.7 g (rural) and 2.9 g (urban) are higher than the mean intake of 2.2 g (5.5 g salt) described in a meta-analysis of data from East African populations (based on few studies, using data from pre-2000) (6), and substantially higher than the sodium consumption described in Malawi in 1986, of 0.86 g (2.2 g salt) in rural areas and 1.65 g (4.1 g salt) in urban areas (27). A 2016 systematic review of daily African sodium intake found a range of 0.67 g of sodium intake per day (∼1.7 g salt) in rural Botswana in the 1960s, to 10.3 g (∼26.4 g salt) in Nigeria in 2006 (7). Overall, Malawi's mean daily sodium consumption fits within the wide range of values described in SSA in recent decades, but appears to have increased markedly since 1986, particularly in rural areas. Although trends in eating habits in Malawi over the past 30 y have been little explored in the literature, it is plausible that both use of salt in household cooking has increased and, even in rural settings, high-salt processed food and flavor enhancers have become much more widely available.

Among people with hypertension, those with a known diagnosis prior to the survey were less likely to have high sodium intake than those with undiagnosed hypertension. Likewise, household members of people with diagnosed hypertension were less likely to have high per capita household plain salt intake or total salt intake than those whose household members had undiagnosed hypertension. As advice is given to reduce salt intake upon diagnosis of hypertension, this could indicate that individuals and households reduce sodium intake when information is provided on its negative health consequences. It is worth noting, however, that this relation could be at least partly explained by residual confounding by an element of socioeconomic status not captured by our data.

During data collection a higher proportion of individuals were missed or refused consent in the urban than in the rural area, which may have introduced some selection bias. Data collection was done during daytimes, so it is likely that those missed were at work. These individuals may have even higher amounts of dietary sodium intake than those included in the study if they have more disposable income allowing them to purchase more processed, high-sodium foods. If so, our study may underestimate population sodium intake, particularly in the urban area. The participants providing urine samples for analysis were not randomly sampled, so it is possible that their sodium intake is not representative of the whole study population. Nonetheless, these participants did come from both urban and rural sites, and as they were selected on 2 nonconsecutive days in the week (whereas participants were recruited for the main study every weekday), they were not clustered in a few geographical locations but spread throughout the study population, so the values are likely to correspond to the range of sodium intakes of the whole population. Household salt use was self-reported based on how long 50 g of salt would typically last in the respondent's household. Although it is possible that participants’ abilities to accurately remember salt purchase frequency may vary (thereby reducing the precision of the per capita household plain salt intake estimates), there is little reason to suspect a differential recall bias.

Our use of spot urine samples will have introduced some error in estimating total dietary sodium intake. To minimize the effect of measurement error on our findings we applied the INTERSALT equation to correct our estimates. Compared with other commonly used equations for deriving 24-h sodium excretion estimates from a spot sample, the INTERSALT equation has been shown to provide the least biased estimates in a racially diverse population in the United States aged 18–39 y (48% African American) (26), although it has not been validated in any African populations (28). Nonetheless, urinary sodium is likely to be an inexact measure of true dietary intake [10% of dietary sodium is thought to be lost through routes other than urine, including through sweat and feces (6)], and the degree of this inexactness is difficult to estimate. Urinary sodium and potassium excretion show seasonal variation in some settings (29–31); however, as the data were collected across all seasons, any seasonal variability should not bias population-level urinary sodium estimates.

Our study was sufficiently large to explore estimates of sodium consumption within age, sex, and location strata. The areas included were broadly typical of rural subsistence farming/fishing communities and rapidly growing socioeconomically mixed urban populations in Malawi (25). As a population survey, it was representative of the adult population of the areas studied (with the exception of young urban men, who represent a large proportion of eligible nonparticipants), and the results are likely to be relevant to other regions within Malawi, and possibly other low-income countries in SSA.

The message is clear that daily sodium consumption exceeds the WHO’s recommended amounts for most of the Malawi adult population, even with our minimum estimate of intake. Both sodium intake and hypertension rates are high in rural as well as urban areas (22), an important factor for planning interventions, as >80% of Malawi's population live in rural areas (24). Given the high salt intake within the home and the practice of eating foods prepared outside the home, particularly in urban dwellers, interventions to reduce sodium intake will need a wide focus and varied approaches to reach all sections of the population, to successfully mitigate hypertension and other complications. Encouragingly, there is a suggestion that on understanding the potential harms of high sodium intake, people are prepared to change their behavior and reduce their sodium consumption.

## Notes

Supported by the Wellcome Trust grant #098610/Z/12/Z (to ACC).
